# The Principles and Practice of Modern Surgery

**Published:** 1844-08-10

**Authors:** 


*The Principles and Practice of Modern Surgery, by* Robert Druitt, Surgeon. *From the third London edition. Illustrated with one hundred and fifty-three wood engravings. With notes and comments.* By Joshua B. Flint, M. D., M. M., S. S., late Professor of Surgery in the Medical Institute of Louisville. Philadelphia, Lea and Blanchard, 1844. 8vo. pp. 550.

The favorable reception of this work by the profession, is evinced by the publication of a third edition in Europe, and now of a second in this country. "The present edition," the author remarks, "is about fifty pages longer than its predecessor. But the additions are solely confined to the practical departments, whilst those chapters which treat of theory, or pathological principles, are rendered somewhat shorter than before."

The notes to the present edition by the American Editor, are sound and practical in their character, although not numerous. The American Publishers, in a note accompanying the work, state that, "prompted by the desire of rendering this work as useful as possible to the student, they have furnished upwards of sixty wood-cuts not contained in the English edition; and which have been selected entirely for the illustration of subjects of practical importance,—principally the surgical anatomy of hernia, and the phenomena and treatment of fractures and dislocations. A few wood-cuts introduced into the third edition, and intended to illustrate pathological conditions, but which they necessarily did imperfectly, from not being coloured, have been omitted,—and some illustrations of the former editions, omitted by the author in the last, being considered useful, have been retained in this."

This is just such a work as is needed by a student in attendance on a course of lectures, being a complete "Manual of Surgery," embracing all the important Surgical diseases, accidents, &c., usually taught in our Medical Schools. We may say, that it is the best adapted for this purpose of any work of the kind with which we are acquainted, and one which it would be well for every medical student to possess. C. H.

